# Pneumatic Dilation in Geriatric Achalasia Patients

**DOI:** 10.5152/tjg.2023.22178

**Published:** 2023-04-01

**Authors:** İlyas Tenlik, Ömer Öztürk, Yasemin Özin, Derya Arı, Orhan Coşkun, Dilara Turan Gökçe, Volkan Gökbulut, Zeynep Göktaş, Zeki Mesut Yalın Kılıç

**Affiliations:** 1 Department of Gastroenterology, Ankara City Hospital, Ankara, Turkey; 2 Department of Nutrition and Dietetics, Hacettepe University Faculty of Health Sciences, Ankara, Turkey

**Keywords:** Achalasia, geriatrics, pneumatic dilatation

## Abstract

**Background:**

The aim of the study was to share the effectiveness of pneumatic dilation in geriatric achalasia patients.

**Methods:**

Achalasia patients over the age of 65 and those under the age of 65 as the control group who received pneumatic dilation as the first-line treatment were evaluated in the study.

**Results:**

The average age of geriatric patients was 72.5 ± 55.92 years (65-90), with 50.3% of them being male. Follow-up was conducted for a mean of 64.52 ± 38.73 months. While pneumatic dilation was successful in 98.6% (141/143) of geriatric patients, it was also successful in 94% (141/150) of non-geriatric patients. Remission after single balloon dilatation was observed in 81.8% of geriatric patients, while it was observed in only 52.7% of non-geriatric patients (*P* = .000). When comparing remission after single dilatation and multiple dilatations, it was observed that geriatric patients who achieved remission after multiple balloon dilatation had higher lower esophageal sphincter pressure and Eckardt scores at the diagnosis and higher lower esophageal sphincter pressure and esophageal body resting pressures after the first balloon dilatation.

**Conclusions:**

The proportion of elders in the world population is increasing daily and this disease has been known to disproportionately afflict this group. Although surgical treatments, in particular per-oral endoscopic myotomy, have recently gained popularity as therapies for achalasia, pneumatic dilation remains the most commonly used in geriatric patients.

**Main Points**

- Pneumatic dilation is a treatment with a very high success rate and acceptable side effect rates in the treatment of geriatric achalasia patients.
- Perforation risk can be minimized thanks to experienced experts, using a 30 mm balloon and low pressure at the beginning and then continuing with gradual dilatations.
- Pneumatic dilation therapy is more successful in the geriatric achalasia group than in non-geriatric patients.

## Introduction

Being the most common primary motor disease of the esophagus, achalasia is characterized by lack of relaxation in the lower esophageal sphincter and loss of peristalsis in the esophageal body. Although it is typically more common in those in their 30s or 60s, achalasia can occur at any age and has an incidence rate of 2-5 per 100 000 persons and prevalence rate of 11-32 per 100 000 persons.^1^ The etiology of achalasia, which is usually idiopathic, is unknown and its most common symptoms include dysphagia, regurgitation, and chest pain. There is no definitive treatment for this disease, with current treatments being palliative. These treatments aim to ease the passage of food from the esophagus to the stomach and to relieve any symptoms by reducing the lower esophageal sphincter pressure (LESP).^2-4^ People are more likely to have multiple comorbidities together with increased average life expectancy. It has been reported that approximately 65% of patients in the geriatric age group have 3 or more comorbid diseases. As the size of geriatric population grows, so does the prevalence and incidence of achalasia. The treatment of achalasia in the geriatric age group is also still controversial.^5^ Current treatment options for achalasia include medical treatment, botulinum toxin injection, endoscopic pneumatic dilation (PD), surgical laparoscopic Heller myotomy (LHM) + Dor fundoplication, and per-oral endoscopic myotomy (POEM).^5-7^ Medical treatments have a low success rate, are administered to patients with advanced comorbidities and a low life expectancy, and have a limited response rate.

They are used as bridge treatments.^6-8^ In terms of non-medical treatments, PD is still the most frequently used treatment today due to its high success rate, ease of administration, applicability to all age groups, ability to be performed under conscious sedation without general anesthesia and without hospitalization, and low cost.^4,6^ In our study, treatment responses to PD in geriatric patients with achalasia were evaluated retrospectively.

## Materials and Methods

### Patients

After the study protocol was reviewed and approved by the ethical committee of the Ankara City Hospital (protocol no: E2-20-26), patient data collection was initiated. The study included achalasia patients over the age of 65 and under the age of 65 as the control group with achalasia who visited the motility laboratory of our gastroenterology clinic between 1995 and 2020. The demographic characteristics and medical records of these patients were reviewed from the files in our motility laboratory and the digital records of our hospital. Diagnosis with achalasia was confirmed by characteristic conventional manometry patterns, barium esophageal radiographs, and/or thorax tomography.^9-11^ Esophagogastroduodenoscopy (EGD) was performed on all patients to exclude organic pathologies and pseudoachalasia. The patients were informed about achalasia and current treatment alternatives. The patients were also informed about the short- and long-term success rates, possible risks and complications of PD, and surgical treatment. The treatment option was chosen by the patients. Patients who initially received PD treatment were included in the study; patients who initially received surgical treatment, patients who had less than 12 months of follow-up, or who had not been followed up on after PD were excluded. Initial symptoms of the patients were calculated using Eckardt score (according to dysphagia, regurgitation, chest pain, weight loss). As classified in previous studies,^12,13^ those with an Eckardt score of 0-3 were considered as symptom free and those with a score of ≥4 were considered as symptoms ongoing. Follow-up and treatment responses were also evaluated using the Eckardt score.

### Pneumatic Dilation

Patients were consciously sedated with midazolam and fentanyl after fasting overnight, and the procedure was performed in the left lateral decubitis posture. After placing the guidewire in the stomach using EGD, a balloon was passed over the guidewire, placed in the esophagogastric junction under fluoroscopy, inflated with 10-15 per square inch (PSI) pressure, and left for 60 seconds.^14^ Following the dilatation, patients were hospitalized for at least 1 day. At 2-4 hours post operation, oral feeding was started for those patients without pain.^9^ However, in patients who had pain or postprandial pain, an oral contrast computed tomography was performed to rule out perforation, and if perforation was detected, the patient was referred to surgery.

In consistent with previous studies,^15^ all dilatations were performed with Rigiflex balloon dilators (30, 35, and 40 mm in a diameter) (Microvasive; Boston Scientific, Watertown, Mass, USA). Gradual dilatation method was used in the patients. Initially, dilatation was performed in all patients with a 30 mm balloon inflated with approximately 10 PSI. After 4-6 weeks of dilation, patients were evaluated using Eckardt scores and manometry. Gradual (35 or 40 mm balloonwith step-wise manner) PD was applied to those with Eckardt scores ≥4 and higher LESP. Patients whose complaints continued despite the third PD or those who refused additional PD were referred to surgery or POEM.

### Statistical Analysis

Statistical analyses were performed using Statistical Package for the Social Sciences version 23.0 (IBM Corp.; Armonk, NY, USA). Kolmogorov–Smirnov normality tests were used to evaluate the distribution of variables. Mann–Whitney *U* and independent samples *t*-test were used for comparison of 2 groups according to normality distribution of variables. A chi-square test was used to assess the relationships between the nominal variables. Data were presented as means ± standard deviation or number and percentage according to their type and distribution. Differences were considered significant at *P*  < .05.

## Results

The study included 143 achalasia patients over 65 years of age and 150 achalasia patients under 65 years of age. Mean age of geriatric patients was 72.7 ± 5.92 (65-90), and 50.3% of them were male. Follow-up was conducted for a mean of 64.52 ± 38.73 months. Mean age of non-geriatric patients was 43.07 ± 11.69 (18-63), and 58.7% of them were male. Follow-up was conducted for a mean of 170.89 ± 75.32 months. When the symptoms at diagnosis were compared, it was found that geriatric patients had more weight loss complaints than non-geriatric patients (*P*  = .01). There was no difference in mean LESP and esophageal body resting pressure at diagnosis between geriatric and non-geriatric patients (*P*  > .5), but the Eckardt score at diagnosis was lower in geriatric patients (*P*  < .000) (Table 1).

In geriatric patients, pneumatic balloon dilatations varying between 1 and 3 sessions (mean ± standard deviation (SD): 1.22 ± 0.49) were performed on 143 patients who underwent balloon dilatation. Although balloon dilatation was successful in 98.6% (141/143) of the patients, it failed in 2 (1.4%) of them. Sustained remission after single balloon dilatation was observed in 81.8% of geriatric patients, while it was observed only in 52.7% of non-geriatric patients. (*P*  < .001). In geriatric patients, sustained remission was observed after the second balloon dilatation in 26 patients and after the third balloon dilatation in 5 patients. Balloon dilatation failed in 2 geriatric patients. The first patient underwent surgery because of perforation after first balloon dilatation. As still being symptomatic 66 months after the first PD, the second patient was administered LHM. Pneumatic balloon dilatations varying between 1 and 5 sessions (mean ± SD; 0.68 ± 0.89) were performed in non-geriatric patients. Although balloon dilatation was successful in 94% (141/150) of the patients, it failed in 9 (6%) of them. One patient underwent surgical treatment due to perforation after first balloon dilatation. In total, 6 of the 8 patients, whose dilatation was unresponsive, underwent LHM and 2 underwent POEM. Perforation developed in 2 of 293 patients in total, and considering that 426 PD dilatations (174 in geriatric group+ 252 in non-geriatric groups) were performed in total, this rate was 0.46% per dilatation (Table 2).

It was observed that geriatric patients who achieved sustained remission after multiple balloon dilatation had higher LESP and Eckardt scores at the time of diagnosis and higher LESP and esophageal body pressures after the first balloon dilatation. Similarly, in non-geriatric patients, LESP at the diagnosis and LESP and esophageal body resting pressure after the first balloon dilation were higher in patients who underwent multiple balloon dilatations. However, there was no significant difference in Eckardt score between those who achieved sustained remission with single or multiple balloons in non-geriatric group (*P*  = .07) (Table 3).

## Discussion

The present study demonstrated that PD is a treatment with a very high success rate and an acceptable side effect profile in the treatment of geriatric achalasia patients. Sustained remission was achieved with single PD in patients with low pre-treatment LESP, low post-treatment LESP and esophageal body resting pressure, and low post-treatment Eckardt scores.

Patients with achalasia are typically treated according to the experience and practices of the clinics. Pneumatic dilation and LHM are treatments that have long been used for achalasia. Therefore, there are sufficient randomized and non-randomized studies on the short- and long-term success rates as well as the side effects of these treatments. Laparoscopic Heller myotomy has a higher success rate in relieving symptoms in the short term. In the long term, the treatment success of LHM is slightly higher than that of repeated gradual PD.^16,17^ In one study,^18^ the remission rate of LHM and PD after 2 years was 89% and 86%, respectively, while at the end of 5 years, this rate was 84% and 82%, respectively. On the other hand, POEM is a treatment that has recently gained popularity for the treatment of achalasia, and its long-term results are still unknown. However, it can only be performed in centers that have adequate experience in advanced therapeutic endoscopic interventions. The 1-year remission rate for POEM was reported to be 93% in one study,^19^ while the 3-year remission rate was reported to be 88.5% in another study.^20^ In a recent meta-analysis by Zhong et al^5^ regarding the role of POEM in geriatric achalasia patients, the clinical success of this treatment was reported to be 95.4% after 1 year and 92.8% after 3 years.

Remission rates after repeated PDs have been reported at different rates in studies.^21-24^ In one study,^25^ success rates of 92%, 84%, 78%, and 64% with 2 or more dilatations were reported in the first, second, third, and fifth years, respectively. In a retrospective study by Zerbib et al^26^ the initial remission rate was 91%, while the 5- and 10-year remission rates of the patients with repeated gradual dilation were reported to be 97% and 93%, respectively. In the present study, the remission rate in the median 64-month follow-up was found to be 98.6% with single or multiple PDs in the geriatric group. On the other hand, in the non-geriatric group, the remission rate was 94% at a median follow-up of 170 months with single or multiple PD. The remission rate in the geriatric patient group was higher than in previous studies that included all age groups. While young patients, particularly male patients under 40, responded less frequently to PD treatment, it was reported that the rate of remission with PD was higher at more advanced ages.^6,9,10,12^ Our study showed that geriatric patients had better PD response rates than non-geriatric patients.

In a retrospective study,^26^ the initial remission rate was found to be 91%, and 5- and 10-year remissions with a single balloon were 67% and 50%, respectively. Similarly, while the success and long-term remission rates of single PD (single dilatation) were found to be between 65% and 69% in various studies, these rates were found to be 77%-94% with repeated dilatations.^26,27-29^ In the present study, in patients who received a single PD, remission rates were found to be 81.8% (117/143) after 5 years in geriatric patients. Single and multiple PD remission rates were higher in this study than in previous studies of the general population. Also, in our study, patients who underwent single balloon treatment had lower basal LESP and Eckardt scores, and their LESPs were lower after dilation. For this reason, it should be kept in mind by physicians that patients who have high LESP at diagnosis and after the first PD may require multiple PDs.

Preliminary dilatation remission rates are important in patients treated with PD, as long-term remissions are greater in those who respond to initial PD treatment. There are also typically lower response rates to repeated PD in those who do not respond initial PD treatment.^6,9^ Müller et al^30^ did not observe any significant difference in Eckardt scores when comparing those who responded to the initial treatment with those who did not. In our study, the Eckardt score at diagnosis in the geriatric group was significantly lower in those who were in remission after single balloon dilatation, but it was not significant in the non-geriatric group. In various studies, PD remission rates have been reported to be lower in those with a high baseline LESP and those who still have a high LESP after PD.^31-33^ In the present study, patients who needed more than 1 PD treatment had higher LESP and Eckardt scores after the first PD in the geriatric group.

In some studies,^34,35^ PD was found to be more effective in female patients whose esophagus was not dilated (moderately dilated esophagus, less than 3 cm). Another study^36,37^ found that having a dilated esophagus (>3 cm) reduced treatment success. In the present study, there was no difference in treatment success based on gender. Also, there was no difference in terms of esophageal dilatation; however, the degree of esophageal dilatation was not determined. When the esophagus was evaluated using EGD, the lumen was stated as dilated or not dilated. Therefore, the actual size of the esophageal lumen diameter was objectively unknown. There was no difference in response to PD between those who had dilated esophageal lumens in their EGD at the time of diagnosis and those who had solid food residuals in their esophagus lumen.

The most serious complication of PD is esophageal perforation (EP), and when PD results from 15 retrospective studies and over 2000 patients were analyzed,^38^ perforation was observed in 3% of cases. In studies conducted with other large patient groups, perforation rates have been reported to be between 1% and 5%.^6,28,39-41^ Ghoshal et al^3^ performed 521 PDs in 433 patients and found that EP was developed in 12 patients (2.7% of patients and 2.3% of dilatations). On the other hand, perforation developed after the first dilatation (30 mm balloon) in only 1 patient, both in the geriatric group and in the non-geriatric group in our study. Perforation developed in 2 of 293 patients (0.68%) in total, and considering that 426 PD dilatations (174 in the geriatric group and 252 in non-geriatric group) were performed in total, this rate was 0.46% per dilatation. Perforations occurred after the first dilatations with 30 mm PD, and surprisingly, we did not observe any perforation after 35 or 40 mm PD. The reasons for our lower rate of perforation may be that the team in this facility has 20 years of experience in this field, and the balloons were inflated with about 10 PSI in the initial 30 mm PD and 12-15 PSI in the repeated dilatations. And, we think perforation risk is higher in the first PD, and gradual PD may decrease the risk of perforation. Microperforations can also be seen after dilatation and can sometimes be overlooked if they are not clinically obvious. In general, it is recommended to evaluate for microperforation under the scope of a water-soluble opaque material after dilatation. As there is no such practice routinely in our clinic, asymptomatic microperforations may have been missed. The other complications such as hemorrhage and intramural hematoma may also occur after PD. No hematoma or active hemorrhage was observed in our patients when the esophagus was checked with endoscopy immediately after PD and no clinically significant hemoglobin decrease was detected in any of the patients during the hospitalization period.

According to some studies,^42-44^ there is a loss of sensory function of the esophagus with age, so elderly achalasia patients tend to be less symptomatic. All geriatric patients in the present study had dysphagia; weight loss was observed in 3-quarters of the patients, and regurgitation symptoms were observed in half of them. It was determined that geriatric patients, in particular, had significantly more weight loss complaints than non-geriatric patients. In light of all these findings, it is believed that there were significant delays in the admission of these geriatric patients to the hospital, as well as delayed diagnoses and treatments.

Our study has some limitations. Despite the fact that the hospital where this research was conducted is a tertiary referral center where the majority of achalasia patients in Turkey are diagnosed and followed up, achalasia typing could not be performed because high-resolution esophageal manometry could not be performed. Reflux that develops after treatment is one of the most serious side effects of achalasia treatments. Patients were not evaluated with a pH meter for reflux after dilation. The duration of symptoms of the patients before the diagnosis was unknown. The esophageal diameters were not recorded in the radiological imaging of the patients’ files, and they were evaluated subjectively because the digital radiology records of our hospital could not be accessed due to technical issues. The dysphagia scores of the patients before and after the treatment could provide valuable information to evaluate the treatment response and in the follow-up of the patients. Unfortunately, it could not be calculated due to the retrospective nature of the study. Also, there were insufficient data about the comorbidities of the patients.

## Conclusion

The proportion of elderly people in the world population is increasing daily, and achalasia has been known to disproportionately affect this group. Although surgical and advanced endoscopic treatments, in particular POEM, have recently gained popularity to treat achalasia, PD has high remission success and a low perforation rate in geriatric patients. Perforation risk can be minimized by using gradual dilatations.

## Figures and Tables

**Table 1.** Characteristic and Demographic Features of Patients

	≥65 years (n = 143)	<65 years (n = 150)	*P*
Mean age at time diagnosis, years, median ± SD (min-max)	72.7 ± 5.92 (65-90)	43.07 ± 11.69 (18-63)	.000
Male, n (%)	72 (50.3)	88 (58.7)	.153
Female, n (%)	71 (49.7)	62 (41.3)	
Median duration time,	64.52 ± 38.73	170.89 ± 75.32	.000
month, median ± SD (min-max)	(12-178)	(12-480)	
Symptoms at diagnosis, n (%)			
Dysphasia	143 (100)	150 (100)	-
Weight loss	105 (73.4)	90 (60)	.01
Retrosternal pain	78 (54.5)	83 (55.3)	.89
Regurgitation	70 (49)	73 (48.7)	.96
Endoscopic finding at diagnosis, n (%)			
Normal esophagus	65 (45.5)	87 (58)	.09
Solid–liquid food residues without esophageal dilation	30 (21)	22 (14.7)	
Dilated esophagus	48 (33.6)	41 (27.3)	
Eckardt score, median ± SD	6.08 ± 1.81 (4-12)	6.93 ± 2.02 (4-12)	.000
LESP at diagnosis, mm Hg, mean ± SD	39.39 ± 17.70	39.21 ± 16.6	.92
Body pressure at diagnosis, mm Hg, mean± SD	11.56 ± 6.27	12.97 ± 8.12	.33
Duration between first and second dilatation, months, median ± SD (min-max)	8.9 ± 11.9 (1-40)	10.4 ± 14.9 (1-72)	.92
Duration between second and third dilatation, months, median± SD (min-max)	4.6 ± 3.9 (1-10)	12.1 ± 15.5 (1-60)	.23

SD, standard deviation; LESP, lower esophageal sphincter pressure.

**Table 2.** Comparison of Balloon Dilatations in Geriatric and Non-geriatric Group

	≥65 years (n = 143)	<65 years (n = 150)	*P*
LESP at diagnosis , mmHg, mean ± SD	39.39 ± 17.70	39.21 ± 16.6	.92
First procedure: balloon diameters used in dilation, n (%)			
30 mm	143 (100)	148 (98.7)	.5
35 mm	0	2 (1.3)	
40 mm	0	0	
First procedure: symptoms after dilatation, n (%)			
No symptoms	87 (60.8)	57 (38)	.000
Decreased symptoms	47 (32.9)	57 (38)	
Stabilized symptoms, no response	9 (6.3)	36 (24)	
LESP after first procedure, mmHg, mean ± SD	12.60 ± 10.49	16.57 ± 10.45	.001
Second procedure: need for dilatation, n (%)			
Yes	26 (18.2)	71 (47.3)	.000
No	117 (81.8)	79 (52.7)	
Duration between first and second dilatation, months, mean ± SD (min-max)	8.9 ± 11.9 (1-40)	10.4 ± 14.9 (1-72)	.92
Second procedure: balloon diameters used in dilation, n (%)			
30 mm	1 (3.8)	6 (8.5)	.76
35 mm	25 (96.2)	64 (90.1)	
40 mm	0	1 (1.4)	
Third procedure: need for dilatation, n (%)			
Yes	5 (3.5)	22 (14.7)	.002
No	138 (96.5)	128 (85.3)	
Duration between second and third dilatation, months mean ± SD (min-max)	4.6 ± 3.9 (1-10)	12.1 ± 15.5 (1-60)	.23
Third procedure: balloon diameters used in dilation, n (%)			
30 mm	1 (20)	0	.22
35 mm	2 (40)	12 (54.5)	
40 mm	2 (40)	10 (45.5)	
Total number of dilatations, mean ± SD	1.22 ± 0.49 (1-3)	1.68 ± 0.89 (1-5)	.000
Surgery after balloon dilatation, n (%)	1 (0.7)	8 (5.3)	.04
Perforation, n (%)	1 (0.7)	1 (0.7)	>.05

LESP, lower esophageal sphincter pressure; SD, standard deviation.

**Table 3.** Comparison of Remission After Single Dilatation and Multiple Dilatations

	≥65 years			<65 years		
	One Dilatation Procedure (*n* = 119)	>1 Dilatation Procedures (*n* = 28)	*P*	One Dilatation Procedure (*n* = 119)	>1 Dilatation Procedures (*n* = 28)	*P*
Age (years), mean ± SD	72.6 ± 5.8	73.1 ± 6.7	>.05	44.9 ± 10.8	40.9 ± 12.3	>.05
Gender (male/female) (%)	49.6/50.4	50.0/50.0	>.05	35.4/64.6	47.9/52.1	>.05
Duration of disease (months), mean ± SD	64.4 ± 39.00	65.1 ± 38.23	>.05	110.1 ± 85.5	105.4 ± 64.09	>.05
Weight loss (kg), mean ± SD	5.7 ± 6.13	5.4 ± 3.95	>.05	4.9 ± 6.49	5.5 ± 5.56	>.05
LESP before treatment, mmHg, mean ± SD	37.2 ± 17.19	49.2 ± 16.93	**.002**	35.3 ± 14.96	43.6 ± 17.32	**.002**
Esophageal body resting pressure before treatment, mean ± SD	11.6 ± 6.49	11.6 ± 5.25	>.05	12.9 ± 8.74	13.0 ± 7.44	>.05
LESP after treatment, mean ± SD	8.9 ± 4.34	29.1 ± 13.83	**<.000**	10.8 ± 6.65	22.9 ± 10.27	**<.000**
Esophageal body resting pressure after treatment, mean ± SD	2.5 ± 3.27	7.8 ± 6.21	**<.000**	1.5 ± 4.75	6.8 ± 6.30	**<.000**
Eckardt score, mean ± SD	5.9 ± 1.79	6.8 ± 1.74	**.003**	6.7 ± 2.02	7.21 ± 1.99	.07

SD, standard deviation; LESP, lower esophageal sphincter pressure.
